# A Prospective, Longitudinal and Exploratory Study of Head and Neck Lymphoedema and Dysphagia Following Chemoradiotherapy for Head and Neck Cancer

**DOI:** 10.1007/s00455-022-10526-1

**Published:** 2022-10-29

**Authors:** Claire Jeans, Bena Brown, Elizabeth C. Ward, Anne E. Vertigan, Amanda E. Pigott, Jodie L. Nixon, Chris Wratten, May Boggess

**Affiliations:** 1 School of Health and Rehabilitation Sciences, The University of Queensland, St Lucia, Australia; 2 Speech Pathology Department, Calvary Mater Newcastle, Locked Mail Bag 7, Hunter Region Mail Centre, NSW 2310 Australia; 3 Centre for Functioning and Health Research, Queensland Health, Buranda, PO Box 6053, Woolloongabba, QLD 4102 Australia; 4 Speech Pathology Department, Princess Alexandra Hospital, Woolloongabba, Australia; 5 Speech Pathology Department, John Hunter Hospital and Belmont Hospital, Locked Bag 1, New Lambton, NSW 2305 Australia; 6 School of Medicine and Public Health, The University of Newcastle, Callaghan, Australia; 7 Centre for Asthma and Respiratory Disease, Hunter Medical Research Institute, New Lambton, Australia; 8 Occupational Therapy Department, Princess Alexandra Hospital, 199 Ipswich Rd, Woolloongabba, QLD 4102 Australia; 9 Radiation Oncology Department, Calvary Mater Newcastle, Locked Mail Bag 7, Hunter Region Mail Centre, NSW 2310 Australia; 10 School of Mathematical and Statistical Sciences, Arizona State University, PO Box 871804, Tempe, AZ 85287-1804 USA

**Keywords:** Head and neck cancer, Radiotherapy, Lymphoedema, Dysphagia, Deglutition, Deglutition disorders, Chemotherapy, Speech pathology

## Abstract

The aim of the study was to examine the following: (a) the trajectory of external and internal head and neck lymphoedema (HNL) in patients with head and neck cancer (HNC) up to 12 months post-chemoradiotherapy (CRT) and (b) the relationship between HNL and swallowing function. Using a prospective longitudinal cohort study, external/internal HNL and swallowing were examined in 33 participants at 3, 6 and 12 months post-CRT. External HNL was assessed using the Assessment of Lymphoedema of the Head and Neck and the MD Anderson Cancer Centre Lymphoedema Rating Scale. Internal HNL was rated using Patterson’s Radiotherapy Oedema Rating Scale. Swallowing was assessed via clinical, instrumental and patient-reported measures. Associations between HNL and swallowing were examined using multivariable regression models. External HNL was prevalent at 3 months (71%), improved by 6 months (58%) and largely resolved by 12 months (10%). In contrast, moderate/severe internal HNL was prevalent at 3 months (96%), 6 months (84%) and at 12 months (65%). More severe penetration/aspiration and increased diet modification were associated with higher severities of external HNL (*p*=0.006 and *p*=0.031, respectively) and internal HNL (*p*<0.001 and *p*=0.007, respectively), and more diffuse internal HNL (*p*=0.043 and *p*=0.001, respectively). Worse patient-reported swallowing outcomes were associated with a higher severity of external HNL (*p*=0.001) and more diffuse internal HNL (*p*=0.002). External HNL largely resolves by 12 months post-CRT, but internal HNL persists. Patients with a higher severity of external and/or internal HNL and those with more diffuse internal HNL can be expected to have more severe dysphagia.

## Introduction

The use of chemoradiotherapy (CRT) treatment regimens have become increasingly used in the treatment of patients with locally advanced head and neck cancer (HNC) [1]. The addition of concurrent chemotherapy, along with enhanced radiotherapy delivery techniques and tumour-related factors have led to improved tumour response and survival rates in patients with HNC [2]. However, despite better management of disease, those treated with CRT continue to experience significant acute and chronic side effects [3, 4], which can be both functionally and psychologically debilitating for those in the survivorship phase of care [5].

Head and neck lymphoedema (HNL) is highly prevalent following HNC treatment [6] and develops when lymph fails to drain through the lymphatic vessels and/or when the lymphatic load exceeds the transport capacity of the lymphatic system [7, 8]. Up to 90% of patients with HNC may be affected by some form of HNL [6] which can develop externally on the soft tissues of the face and neck, internally within the oral cavity, pharynx or larynx, or as a combination of both [9]. The development of HNL has historically been attributed to surgical treatments, including the removal of lymphatic structures, vessels and nodes. However, the presence of significant tumour bulk and management via non-surgical treatment options, such as high-dose radiotherapy and concurrent chemotherapy, may also damage the lymphatic structures and disrupt the normal flow of lymph fluid [10, 11].

It has been shown that patients are less likely to experience HNL with increasing time post-treatment [11]. This notion is consistent with the anecdotal view that HNL will eventually resolve with conservative management. However, recent studies have shown that HNL may not completely resolve [6, 12]. Ridner et al. [6] prospectively examined 83 HNC patients who had undergone some form of multimodal treatment and found that HNL was most prevalent 3 months post-treatment, with 90% of the cohort experiencing external HNL and 86% internal HNL. The severity of both external and internal HNL appeared to gradually improve from 12 to 18 months. However, 82% and 80% still had some degree of external and internal HNL at the completion of the study. Tribius et al. [12] reported similar results in a longitudinal study of 280 HNC patients who had also undergone surgery and postoperative radiotherapy (+/− chemotherapy). In their cohort, 80% had some degree of external and/or internal HNL at 3 months post-treatment, and 38% continued to have some degree of HNL at the end of the observation period (time varies).

The high prevalence of HNL in patients following HNC treatment is concerning as HNL has been associated with a wide range of physical, functional and psychological issues [13–17]. In particular, patients who have more severe external and internal HNL have been shown to experience increasing dysphagia [13, 16–19] and have more severe laryngeal penetration and/or aspiration, experience more pharyngeal residue, require increased diet modification and have more self-reported symptom burden in relation to eating solid foods [13, 16–19]. However, the relationship between HNL and dysphagia has only been examined in cross-sectional samples with heterogeneous populations, and it is unclear if the relationship remains at different time points post-treatment. The primary aim of this study was therefore to explore the trajectory of external and internal HNL in patients with HNC who have been treated with CRT and describe the changes in HNL location and severity over a 12-month period. It also aimed to explore the relationship between external and internal HNL and the presence of dysphagia, including penetration–aspiration status, functional diet status and patient-reported swallowing outcomes during this time period.

## Material and Methods

A longitudinal cohort study design was used. It forms part of a larger study that has been described in further detail elsewhere [16]. Ethics approval was obtained from Hunter New England Human Research Ethics Committee (15/02/18/4.07), University of Queensland Medical Research Ethics Committee (2015000362) and Calvary Mater Newcastle Research Governance Unit (SSA/15/HNE/45).

### Participants

Participants were prospectively recruited from the Radiation Oncology Clinic at the Calvary Mater Hospital Newcastle, Australia, using a convenience sampling strategy. Recruitment occurred from September 2015 to July 2018, and follow-up continued until November 2019. Eligibility criteria included the following: (1) new diagnosis of oral, nasopharyngeal, oropharyngeal, laryngeal or hypopharyngeal cancer; and (2) planned to receive curative CRT. Exclusion criteria included the following: (1) treated with palliative intent; (2) recurrent or metastatic disease; (3) pre-existing comorbidity conditions that may result in HNL (e.g. trauma), or impact swallowing, voice or speech function (e.g. neurological injury or insult); or (4) unable to provide informed consent.

### Procedure

Measures were collected at 3, 6 and 12&nbsp;months post-treatment. A chart review was undertaken for each participant prior to each time point to ensure ongoing disease-free status. External HNL, internal HNL and swallowing were assessed at each time point.

### Assessment of Head and Neck Lymphoedema

External HNL was assessed with two assessments. It was primary graded with the MD Anderson Cancer Centre (MDACC) Lymphoedema Rating Scale [8] which uses 5 points to capture HNL across the continuum of soft swelling to fibrosis. For the purposes of this study, 0 was classified as normal, 1a mild, 1b moderate, 2 severe and 3 profound external HNL. The secondary assessment included the Assessment of Lymphoedema of the Head and Neck (ALOHA) [20] which utilises surface tape measurements and the tissue dielectric constant (TDC) measured with a MoisureMeterD (MMD; Delfin Technologies Ltd, Kuopio, Finland). The participant's weight is also recorded. The standardised setup positioning protocol was applied and tape measurements were taken at the lower neck circumference, upper neck circumference and length from ear to ear. For the TDC measure, the 2.5-mm MMD probe was placed on the skin surface 8 cm below the lower lip edge (3 measurements taken). Both the ALOHA tape measurement system and TDC measure have demonstrated strong inter-rater reliability [21]. Normative values for the TDC [22] were also used as reference data. It was routine clinical care to refer patients onto the physiotherapy department for further assessment and management when external HNL was identified.

Internal HNL was assessed via transnasal laryngoscopy. The presence, location and severity of internal HNL was rated with Patterson’s Radiotherapy Oedema Rating Scale [23] (scale recently revised [24]). This scale includes 13 laryngopharyngeal sites and ratings of normal, mild, moderate or severe are available to rate each site. The scale has shown moderate agreement for inter-rater reliability and very good agreement for intra-rater reliability [23]. To further assist with rating determinations, an education package was developed which included images of HNL at different severity levels and at each site. Twenty percent of the total recordings were re-rated by the primary investigator at least 3 months after the initial rating, and a second speech pathologist to assess intra- and inter-rater reliability.

In addition to the 13 site ratings, three internal HNL summary variables were also generated: (1) a ‘maximum severity score’ which reflected the maximum severity rating obtained across the 13 internal sites; (2) a ‘sum severity score’ which was generated by allocating each severity rating a score (i.e. normal = 0, mild = 1, moderate = 2, severe = 3) and adding the scores across the 13 internal sites; and (3) the ‘number of internal sites affected by HNL’ was generated by counting the number of internal sites identified as having HNL (three severity variations—any severity; moderate or severe; or severe). The ‘maximum severity score’ and ‘number of internal sites affected by HNL’ have previously been used in the literature [6, 9, 16, 18]. The ‘sum severity score’ was novel.

### Assessment of Swallowing

The swallowing assessment included clinical, instrumental and patient-reported outcome measures. The Mann Assessment of Swallowing Ability—Cancer (MASA-C) [25] was used to grade oral musculature, cranial nerve and clinical swallowing ability. It has a maximum score of 200 which indicates swallowing within normal limits, whilst a score of 185 or less indicates the presence of dysphagia [25]. The Functional Oral Intake Scale (FOIS) [26] was used to grade functional diet status. The FOIS classifies diet status as non-oral (scores 1–3) or oral (scores 4–7) and considers the number of diet consistencies tolerated, and the need for special preparations or compensations.

For instrumental assessment, a fiberoptic endoscopic evaluation of swallowing (FEES) was performed. Participants’ swallowed two mouthfuls of blue dyed water, the size of which were self-determined, and the Penetration–Aspiration Scale (PAS) [27] was used to grade laryngeal penetration and aspiration events on the worse of the two swallows. PAS scores of 1–2, indicating that *material does not enter the airway* or *material enters the airway, remains above the true vocal folds and is ejected*, were considered normal. Scores of 3–8 were considered dysfunctional [28]. Twenty percent of the total FEES recordings were re-rated as per the reliability protocol described above.

Finally, the Vanderbilt Head and Neck Symptom Survey (VHNSS) (v2.0) Plus General Symptom Scale [29] was used to examine self-perceived symptom burden in relation to swallowing ability and nutritional status. It was given to participants to independently complete and return. VHNSS questions use a Likert scale where a score of 0 indicates no symptoms and 10 indicates severe symptoms. Consistent with prior research [29], questions from four symptom subscales, the *swallow general* (questions 5–13), *swallow solids* (questions 5, 7, 8 and 10), *swallow liquids* (questions 6 and 9) and *nutrition* (questions 1–4), were summed and then collapsed and classified as mild (scores 1–3), moderate (scores 4–6) and severe (7–10).

At the study centre, it was routine clinical care for all participants to receive regular speech pathology assessment and management for their swallowing, voice and speech function throughout their CRT, and up to 3 months post-treatment. After this time, review was only provided on an as-needed basis.

### Statistical Methods

Analyses were conducted by the primary investigator (CJ) and study statistician (MB) using the statistical software package Stata 16 [30]. Cohen’s kappa coefficient was used to assess the intra- and inter-rater reliability of the Patterson Radiotherapy Oedema Rating Scale and PAS ratings, which were observed via the transnasal laryngoscopy and FEES assessments (20% subsample). Linear weights (*Kw*) were applied [31] and the strength of agreement was classified as slight (*Kw* 0–0.20), fair (*Kw* 0.21–0.40), moderate (*Kw* 0.41–0.60), substantial (*Kw* 0.61–0.80) and almost perfect (*Kw* 0.81–1) [32].

Paired *t*-tests were used to examine the changes in continuous variables between time points (i.e. 3 vs. 6 months and 6 vs. 12 months). Wilcoxon signed-rank test was used to examine the changes in categorical variables between time points. Linear regression models were used to examine the associations between time points in the ALOHA tape measurement system and changes in weight. Shapiro–Wilk was used to assess the normality of residuals.

Regression models were used to examine associations between the swallowing and the HNL variables. A model was fitted for each HNL and swallowing variable (i.e. 6 × 17 models—all reported in Table 1). Linear regression models were used to examine the relationships between the HNL variables (explanatory) and the FOIS, MASA-C and VHNSS subscale scores (response). Logistic regression models were used to examine the relationships between the HNL variables (explanatory) and PAS scores (response), since 56% of the total observations (*n* = 48/86) had a normal PAS score (i.e. scores 1–2). Backwards stepwise methodology was employed during the fitting of these models to account for possible effects of other variables, such as age, tumour and nodal stage, and external HNL treatment. Data from the 3, 6 and 12 month time points were merged to create one main dataset (i.e. 86 observations on 33 participants). This was undertaken due to the small sample size at each time point and the low variability of the HNL and swallowing outcomes (i.e. most outcomes were tightly clustered around the mean). Clustered standard errors were used in the models, since more than one observation was made on each participant and this accounted for the time point variable [33]. Significance was set at *p* < 0.05.

**Table 1:** Relationships between swallowing outcomes and HNL via regression modelling

Response variables (possible range)	PAS (1–2 vs. 3–8)^a^		FOIS (1–7)^b^		MASA-C (40–200)^2^		VHNSS solids (0–50)^2^		VHNSS liquids (0–20)^2^		VHNSS nutrition (0–40)^2^	
Explanatory variables	Odds ratio	*p*	*β*	*p*	*β*	*p*	*β*	*p*	*β*	*p*	*β*	*p*
External HNL^c^	3.5	**<0.01**	− 0.7	**0.04**	− 9.4	**<0.01**	4.5	**<0.01**	1.8	**0.04**	3.7	**0.05**
Max internal HNL	5.2	**<0.01**	− 0.4	**0.01**	− 5.6	**<0.01**	2.1	0.19	− 0.3	0.44	0.4	0.76
Sum internal HNL	1.2	**<0.01**	− 0.1	**0.01**	− 0.7	**<0.01**	0.4	**0.02**	0.0	0.46	0.1	0.35
Number of internal sites	1.6	**0.04**	− 0.2	**<0.01**	− 1.7	**<0.01**	1.1	**<0.01**	0.3	**0.04**	0.7	**0.03**
Individual internal sites												
Base of tongue	1.2	0.57	0	0.95	− 1.5	0.27	− 1.0	0.41	− 0.3	0.17	0.2	0.86
Posterior pharyngeal wall	2.1	0.07	− 0.2	0.24	− 2.4	0.12	1.2	0.43	− 0.6	0.15	− 0.5	0.70
Epiglottis	2.5	**<0.01**	− 0.3	0.10	− 3.6	**0.02**	2.3	0.07	− 0.3	0.53	− 0.2	0.87
Pharyngoepiglottic folds	2.4	**<0.01**	− 0.4	**0.02**	− 4.5	**0.01**	2.5	**0.02**	0.1	0.87	0.7	0.49
Aryepiglottic folds	4.3	**<0.01**	− 0.6	**<0.01**	− 6.6	**<0.01**	3.0	**0.02**	0.5	0.22	1.7	0.12
Interarytenoid space	2.3	**0.01**	− 0.5	**0.01**	− 5.5	**0.01**	3.1	**0.01**	0.5	0.22	1.2	0.18
Cricopharyngeal prominence	3.3	**<0.01**	− 0.5	**0.01**	− 5.9	**<0.01**	3.4	**<0.01**	0.6	0.09	1.5	0.14
Arytenoids	3.4	**<0.01**	− 0.4	**0.03**	− 5.2	**0.01**	3.4	**0.01**	0.6	0.12	1.6	0.25
False vocal folds	2.3	0.05	− 0.3	0.09	− 4.9	**0.01**	2.8	**0.02**	0.5	0.12	1.2	0.34
True vocal folds	1.9	0.33	− 0.8	0.28	− 13	0.18	4.1	0.61	4.0	0.28	− 3.6	0.49
Anterior commissure	2.8	−	−	0.52	− 3.5	0.10	0.2	0.86	0.3	0.56	− 0.1	0.96
Valleculae	1.8	0.14	− 0.2	0.15	− 3.1	**0.02**	− 0.5	0.62	− 0.5	0.16	0.7	0.45
Pyriform sinus	3.9	**<0.01**	− 0.5	**0.01**	− 5.8	**<0.01**	3.2	**<0.01**	0.4	0.29	0.7	0.41

*PAS* penetration-aspiration scale, *FOIS* functional oral intake scale, *MASA-C* mann assessment of swallowing ability—Cancer, *VHNSS* vanderbilt head and neck symptom survey, *p* = *p*-value

Bold type indicates statistical significance *p* < 0.05

^a^Logistic regression with response PAS=1 or 2, with standard errors clustered on participant

^b^Linear regression, with standard errors clustered on participant

^c^Determined by the MDACC Scale rating only

## Results

**Fig. 1:**
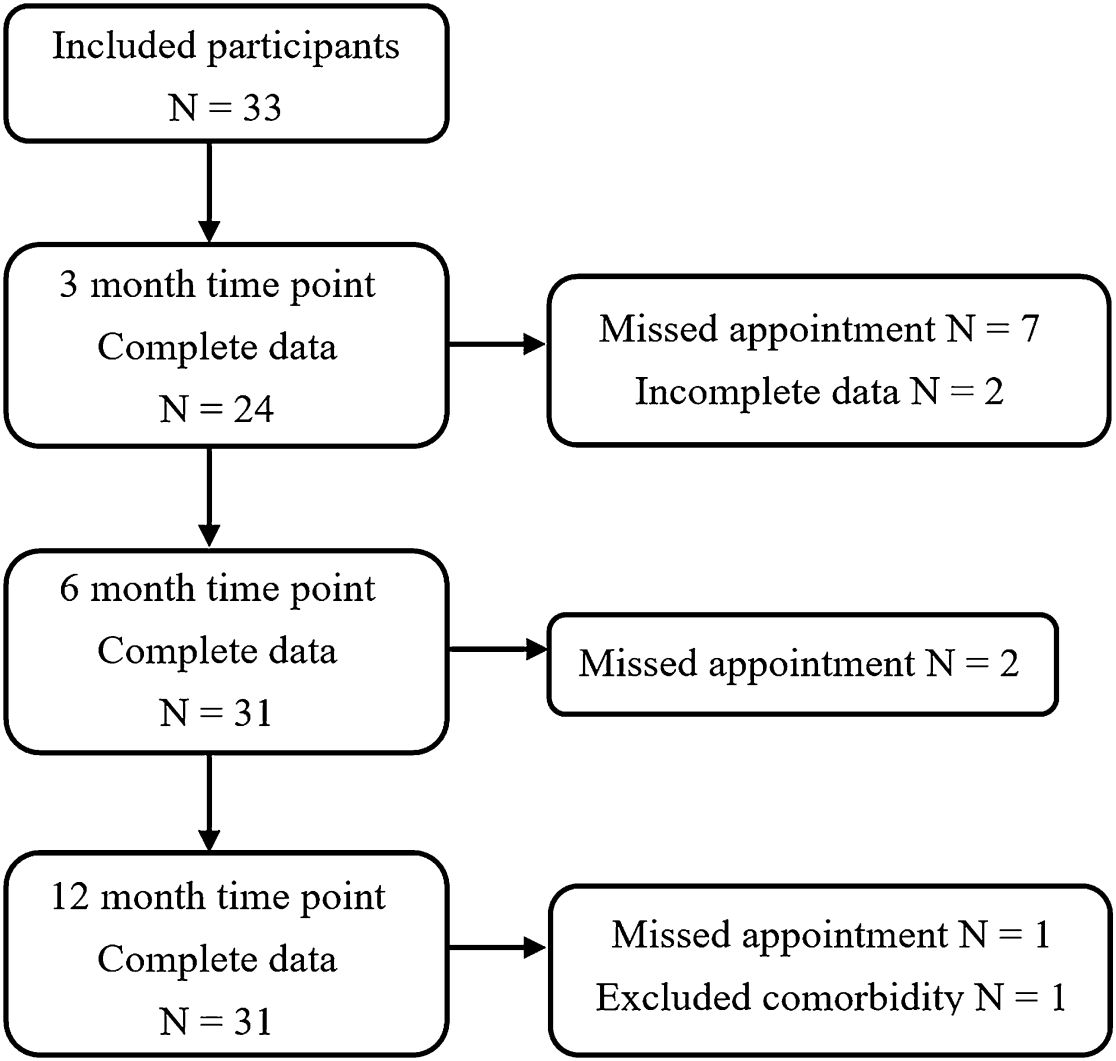
Number of participants by time point. Recruitment occurred at the start of CRT. Participation in the 3-month time point was low. Participants often declined this study appointment due to the recency of treatment and some anxiety surrounding their post-treatment PET-CT scan results. In such cases, their first participation was at 6 months

Forty-two participants were recruited at the start of CRT (Fig. 1). There was attrition of two participants due to recurrent/residual disease and three participants due to complications with comorbidities. Only participants with complete data for at least two of the three follow-up time points were included in the analysis, which necessitated the removal of four participants who only had data at one time point. Of the remaining 33 participants, 20 had complete data at all three time points, and 13 had complete data at two time points. There were a total of 86 observations (*n* = 24 at 3 months, *n* = 31 at 6 months and *n* = 31 at 12 months). There was an overall attrition rate of 21%, and participation rates of 73% at 3 months and 94% at 6 and 12 months. Note that participants remained in the study if a time point was missed.

Participants were predominantly male and under 65 years of age (Table 2). The vast majority presented with early-stage oropharyngeal tumours had advanced nodal metastases and human papilloma virus (HPV)-positive disease. All participants were treated with intensity-modulated radiation therapy (IMRT) or volumetric-modulated arc therapy (VMAT). Most received conventional radiation treatment of 70 Gy/35# and three cycles of high-dose cisplatin. All participants were disease free at the time of participation.

**Table 2:** Demographic, disease and treatment data

Characteristic		*n* = 33
Age (years) (mean (SD))		59.9 (7.7)
		% (n)
Gender	Male	91 (30)
	Female	9 (3)
Primary site	Nasopharyngeal	3 (1)
	Oropharyngeal	91 (30)
	Laryngeal	6 (2)
T classification	T 1–2	67 (22)
	T 3–4	30 (10)
	T X	3 (1)
N classification	N 0	6 (2)
	N 1	6 (2)
	N 2–3	88 (29)
TNM staging	Stage 0	3 (1)
	Stage I	3 (1)
	Stage III	9 (3)
	Stage IVA	82 (27)
	Stage IVB	3 (1)
HPV status	Positive	76 (25)
	Negative	24 (8)
Radiation treatment	70Gy/35#	94 (31)
	Other	6 (2)
Chemotherapy	Cisplatin	70 (23)
	Cetuximab	24 (8)
	Other	6 (2)
Baseline FOIS	Levels 4–6	27 (9)
	Level 7	73 (24)

Percentages may not total 100 due to rounding

*T* tumour, *N* nodal, *HPV* human papillomavirus, *SD* standard deviation, *FOIS* functional oral intake scale

### Intra- and Inter-rater Reliability: Patterson Radiotherapy Oedema Rating Scale and Penetration–Aspiration Scale

Eighteen of the 86 transnasal laryngoscopy and FEES assessments were re-rated. The intra-rater reliability of the Patterson Radiotherapy Oedema Rating Scale ratings (13 internal sites) was on average, substantial (*Kw* = 0.83). The sites with the highest agreement (almost perfect) were the true vocal folds, cricopharyngeal prominence and posterior pharyngeal wall (*Kw* = 1, 0.95, 0.91, respectively). The sites with the lowest agreement (substantial) were the false vocal folds, anterior commissure and interarytenoid space (*Kw* = 0.60, 0.74, 0.74, respectively). The intra-rater reliability for the PAS ratings was perfect (*Kw* = 1).

The inter-rater reliability of the Patterson Radiotherapy Oedema Rating Scale ratings was on average, moderate (*Kw* = 0.44). The sites with the highest agreement (substantial) were the aryepiglottic folds, epiglottis and cricopharyngeal prominence (*Kw* = 0.69, 0.68, 0.64, respectively). The sites with the lowest agreement (slight) were the true vocal folds, base of tongue and false vocal folds (*Kw* = 0, 0.12, 0.15, respectively). The inter-rater reliability for the PAS was substantial (*Kw* = 0.78).

### Head and Neck Lymphoedema: General Presentation

All participants had some form of HNL at each time point (Table 3). At 3 months, the majority (71%) had combined external and internal HNL, and the majority (58%) continued to have combined HNL at 6 months. However, by 12 months, most participants had internal HNL only (90%).

**Table 3:** Head and neck lymphoedema prevalence and outcome measures by time point

HNL type	3 mths, *n* = 24	6 mths, *n* = 31	12 mths, *n* = 31	*p, *3 vs. 6 mths	*p, *6 vs. 12 mths
No HNL	0	0	0		
Both external and internal HNL^a^	71 (17)	58 (18)	10 (3)		
Internal HNL only	29 (7)	42 (13)	90 (28)		
External HNL only^a^	0	0	0	0.059	**<0.001**^b^
External HNL outcome measures					
MDACC Scale (% (n))					
No visible oedema (0)	29 (7)	42 (13)	90 (28)		
Soft visible oedema (1a)	63 (15)	48 (15)	6 (2)		
Soft pitting oedema (1b)	8 (2)	10 (3)	3 (1)	0.089	**<0.001**^b^
TDC (Mean (SD))	29.8 (8.7)	27.7 (9.6)	21.3 (8.8)	0.167	**0.002**^c^
Tape measurements (cm) (Mean (SD))					
Lower neck	42.3 (4.6)	41.6 (3.7)	40.5 (4.6)	**0.005**	0.151^c^
Upper neck	44.9 (5.2)	43.6 (4.5)	42.2 (4.9)	**0.019**	0.220^c^
Ear to ear	25.5 (1.9)	26.0 (2.7)	25.1 (2.1)	**0.028**	**0.032**^b^
Weight (kg) (Mean (SD))	74.9 (16.1)	76.3 (14.4)	80.3 (12.4)	0.903	**0.010**^b^
External HNL treatment (% (n))					
Yes	13 (3)	26 (8)	10 (3)		
No	88 (21)	77 (24)	90 (28)	0.083	0.059^b^
Internal HNL outcome measures					
Maximum severity (all sites) (% (n))					
Normal	0	0	0		
Mild	4 (1)	16 (5)	35 (11)		
Moderate	38 (9)	48 (15)	48 (15)		
Severe	58 (14)	35 (11)	16 (5)	**0.011**	**0.013**^b^
Sum severity (all sites)					
Mean (SD)	18.9 (5.9)	15.8 (7.7)	9.7 (6.5)		
Range	10–28	6–32	2–29	**0.009**	**<0.001**^b^
Number of sites (any severity)					
Mean (SD)	10.5 (1.5)	9.9 (2.2)	7.0 (2.9)		
Range	7–12	5–13	2-12	**0.022**	**<0.001**^c^
Number of sites (moderate/severe)					
Mean (SD)	6.4 (3.1)	4.5 (4.0)	2.2 (2.9)		
Range	0–11	0–13	0–10	**0.017**	**<0.001**^c^
Number of sites (severe)					
Mean (SD)	2.0 (2.2)	1.4 (2.3)	0.5 (1.4)		
Range	0–6	0–9	0–7	0.053	**0.007**^c^
Internal site severity (Mod or Severe % (n))					
Arytenoids	67 (16)	52 (16)	29 (9)	0.083	**0.007**^b^
Epiglottis	63 (15)	52 (16)	35 (11)	**0.046**	0.317^b^
Pharyngoepiglottic folds	63 (15)	45 (14)	26 (8)	**0.020**	**0.002**^b^
Aryepiglottic folds	67 (16)	42 (13)	19 (6)	**0.020**	**<0.001**^b^
Valleculae	67 (16)	26 (8)	10 (3)	**0.003**	**0.004**^b^
Posterior pharyngeal wall	54 (13)	58 (18)	29 (9)	0.739	**0.001**^b^
Interarytenoid space	58 (14)	42 (13)	23 (7)	0.096	**0.006**^b^
Pyriform sinus	50 (12)	29 (9)	10 (3)	**0.003**	**<0.001**^b^
Base of tongue	42 (10)	35 (11)	23 (7)	0.698	**0.006**^b^
Cricopharyngeal prominence	58 (14)	35 (11)	16 (5)	**0.002**	**0.010**^b^
False vocal folds	29 (7)	26 (8)	0	0.364	**<0.001**^b^
Anterior commissure	17 (4)	10 (3)	3 (1)	0.216	**0.021**^b^
True vocal folds	4 (1)	3 (1)	0	0.583	0.317^b^

Percentages may not total 100 due to rounding

*HNL* head and neck lymphoedema, *MDACC* MD Anderson cancer centre, *TDC* tissue dielectric constant, *mths* months, *SD* standard deviation, *p* *p*-value

^a^Determined by the MDACC Scale rating only

^b^Wilcoxon signed-rank test

^c^Paired *t*-test

### External Head and Neck Lymphoedema: Prevalence, Location and Severity

The MDACC Scale demonstrated that external HNL was most prevalent at 3 months (71%), with a small improvement at 6 months (58%) and a large improvement at 12 months (10%) (Table 3). There was a significant reduction in the MDACC Scale score between 6 and 12 months (*z* = 3.873, *p* < 0.001), but not between 3 and 6 months (*z* = 1.698, *p* = 0.089). When external HNL was present across the three time points, it was largely considered mild (MDACC Scale rating 1a). Descriptive analysis also revealed the most frequently involved site was the submental region, which was affected in all participants who had external HNL at 3 and 12 months, and all but one participant at 6 months.

Both statistical and clinical improvements were also evident in the ALOHA’s TDC value and the tape measurements of the upper and lower neck circumferences (Table 3). Significant improvements in the TDC were found between 6 and 12 months (*t*_*26*_ = − 3.376, *p* = 0.002, 95% confidence interval (CI) = [− 10.0, − 2.4], although still slightly outside the normal range at 12 months [22]), but not between 3 and 6 months (*t*_*19*_ = − 1.436, *p* = 0.167, 95% CI = (− 7.2, 1.3)). In contrast to the MDACC Scale and TDC, significant improvements in the tape measurement of the upper and lower neck circumference were found between 3 and 6 months (*t*_*19*_ = − 2.553, *p* = 0.019, 95% CI = (− 3.0, − 0.3) and *t*_*19*_ = − 3.142, *p* = 0.005, 95% CI = (− 1.8, − 0.4), respectively), but not between 6 and 12 months (*t*_*27*_ = 1.254, *p* = 0.220, 95% CI = (− 0.8, 3.2) and *t*_*27*_ = 1.478, *p* = 0.151, 95% CI = (− 0.4, 2.7), respectively). However, between 6 to 12 months, an increase in body weight was significantly related to an increase in upper and lower neck circumferences (*F(1,26)* = 6.07, *p* = 0.021 and *F(1,26)* = 7.16, *p* = 0.013, respectively), an effect unseen in the other external HNL measurements.

Although the general trend was for external HNL to improve in the study cohort, individual case analysis revealed that 6% of the total cohort had a worse MDACC Scale score between 3 and 6 months. Nine percent also had a worse TDC between 3 and 6 months, whilst 3% has a worse TDC between 6 and 12 months.

All participants who presented with external HNL at 3 months were referred to the physiotherapy department for further assessment and management. However, only 39% of participants who had ongoing external HNL at 6 months had received external HNL therapy (Table 3). Of the three participants who still had external HNL at 12 months, all had previously received treatment and were continuing self-management at home.

### Internal Head and Neck Lymphoedema: Prevalence, Location and Severity

All participants had some degree of internal HNL at each time point (Table 3). The maximum severity score indicates that most participants had moderate or severe internal HNL at 3 months (96%), 6 months (84%) and 12 months (65%). The number of internal sites affected by moderate or severe internal HNL also improved over time. At 3 months, participants had on average 6.4 of 13 internal sites that were moderate or severe, but this reduced to 4.5 sites at 6 months, and 2.2 sites at 12 months. Significant improvements in the maximum severity score and number of internal sites affected by moderate or severe HNL were found between 3 and 6 months (*z* = − 2.530, *p* = 0.011 and *t*_*21*_ = − 2.584, *p* = 0.017, 95% CI = (− 3.1, − 0.3), respectively), and 6 and 12 months (*z* = − 2.496, *p* = 0.013 and *t*_*28*_ = − 4.838, *p* < 0.001, 95% CI = (− 2.5, − 1.0), respectively).

Once again, whilst the general trend was for internal HNL to improve, 12% of the total cohort had an increased number of internal sites involving HNL between 3 and 6 months, and 3% between 6 and 12&nbsp;months. Nine percent also had a worse sum severity score between 3 and 6 months, and 3% had a worse maximum severity score between 3 and 6 months.

**Fig. 2:**
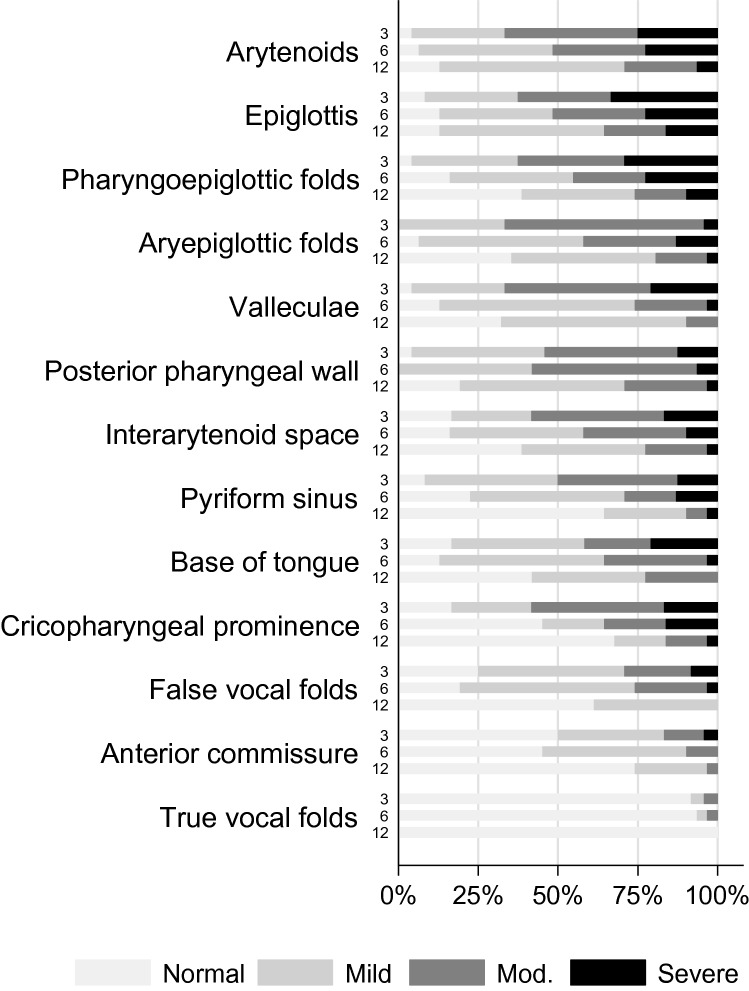
Location and severity of internal head and neck lymphoedema by time point

The location and severity of internal HNL at individual sites is shown in Fig. 2. Of note, only five of 13 individual sites significantly improved (*p* < 0.05) between both 3 and 6 months and 6 and 12 months. These included the cricopharyngeal prominence, pyriform sinus, valleculae, pharyngoepiglottic folds and the aryepiglottic folds.

### Swallowing Outcomes

A third of the cohort had a dysfunctional PAS score (i.e. scores 3–8) at 3 months, indicating some degree of laryngeal penetration or aspiration with thin fluids during the FEES assessment (Table 4). By 12 months, only 13% had a dysfunctional PAS score. Ninety-two percent scored 185 or less on the MASA-C at 3 months, indicating the presence of dysphagia; but by 12 months, only 55% scored 185 or less. Most participants (79%) required some form of diet modification (FOIS scores 4–6) at 3 months, and 68% still required diet modification at 12 months. Seventy-four (of the total 86) VHNSS questionnaires were returned from 32 participants. The presence and severity of individual symptoms are reported in Table 4.

**Table 4:** Swallowing outcome measures by time point

Outcome measures	3 mths, *n* = 24, % (*n*)	6 mths, *n* = 31, % (*n*)	12 mths, *n* = 31, % (*n*)	*p, *3 vs. 6 mths	*p, *6 vs. 12 mths
PAS					
Level 1-2	67 (16)	65 (20)	87 (27)		
Levels 3-6	21 (5)	16 (5)	13 (4)		
Levels 7-8	13 (3)	19 (6)	0	0.317	0.077^a^
FOIS					
Level 1	4 (1)	3 (1)	0		
Levels 2-3	0	0	0		
Levels 4-6	83 (20)	74 (23)	68 (21)		
Level 7	13 (3)	23 (7)	32 (10)	**0.046**	0.180^a^
MASA-C					
Mean (SD)	177 (9.2)	178 (13.7)	186 (7.6)		
Range	154-187	120-192	174-198	0.450	**0.001**^b^
VHNSS subscale questions (Mean (SD))	*n* = 20	*n* = 27	*n* = 27		
Swallow general					
Longer to eat due to swallowing (q13)	5.2 (3.4)	5.1 (4.0)	3.9 (3.2)	0.264	0.423^b^
Swallowing takes great effort (q12)	3.0 (2.3)	2.9 (2.7)	2.2 (2.1)	0.627	0.418^b^
Swallow solids					
Trouble eating certain solid foods (q5)	6.2 (2.9)	5.3 (3.4)	4.1 (3.1)	0.083	0.204^c^
Food stuck in mouth (q7)	3.3 (2.4)	3.0 (2.5)	2.1 (1.5)	0.937	0.024^c^
Food stuck in throat (q8)	3.0 (2.5)	4.0 (3.0)	2.6 (2.1)	0.667	0.083^c^
Cough after swallow (q11)	2.4 (2.3)	2.3 (2.4)	1.7 (1.8)	0.097	0.259^c^
Choke or strangle on solid foods (q10)	1.8 (1.9)	2.6 (2.6)	1.4 (0.8)	1.000	0.136^c^
Swallow liquids					
Choke or strangle on liquids (q9)	1.7 (1.7)	1.7 (1.8)	1.1 (0.5)	0.054	0.188^c^
Trouble drinking thin liquids (q6)	1.6 (2.0)	2.3 (2.4)	1.0 (0.2)	0.370	**0.014**^c^
Nutrition					
Lost appetite (q2)	3.6 (2.3)	3.2 (3.3)	2.0 (2.2)	0.685	**0.004**^c^
Liquid supplements to maintain weight (q3)	3.6 (3.1)	2.3 (2.2)	1.6 (1.4)	0.064	0.343^c^
Trouble maintaining weight due swallow (q4)	3.1 (2.6)	2.0 (2.3)	1.3 (0.9)	0.108	0.219^c^
Losing weight (q1)	3.0 (2.9)	2.4 (2.1)	1.5 (1.1)	0.149	0.113^c^

Percentages may not total 100 due to rounding* mths* months, *SD* standard deviation, *PAS* penetration-aspiration scale, *FOIS* functional oral intake scale, *MASA-C* mann assessment of swallowing ability—cancer, *VHNSS* vanderbilt head and neck symptom survey, *VHNSS scoring* 1–3 mild, 4–6 moderate, 7–10 severe, *p* = p-value

^a^Wilcoxon signed-rank test

^b^Paired t-test

### Associations Between External and Internal Head and Neck Lymphoedema and Swallowing Outcomes

The relationships between the external and internal HNL variables and swallowing outcomes are presented in Table 1. Backwards stepwise procedures did not identify any other variables of significance, and there was no loss of normality in model residuals. Regression modelling revealed significant (*p* < 0.05) relationships between dysfunctional PAS scores (i.e. scores 3–8), FOIS scores and MASA-C scores and external HNL. Significant relationships (*p* < 0.05) were also found between dysfunctional PAS scores, FOIS scores and MASA-C scores and all three of the internal HNL summary variables. These results indicate that participants were more likely to experience dysphagia, have laryngeal penetration and/or aspiration and require increased diet modification if they had a higher severity of external and/or internal HNL, and if they had more diffuse internal HNL. Of interest, internal HNL that occurred at the aryepiglottic folds, pyriform sinus and cricopharyngeal prominence were also amongst some of the strongest predictors of dysfunctional PAS scores, worse FOIS scores and worse MASA-C scores.

The VHNSS *swallow general* and *swallow solids* subscales were highly significantly correlated (*r* = 95%). Therefore, only the VHNSS *swallow solids, swallow liquids and nutrition* subscales were examined in the regression modelling. In these models, there was less association between the VHNSS subscales and the HNL variables, as compared to the PAS, FOIS and MASA-C scores. But of note, significant relationships were found between the *swallow solids* subscale and external HNL, the sum severity score for internal HNL and the total number of internal sites affected by HNL (all *p* < 0.05). These results signify that participants with a higher severity of external HNL and more diffuse internal HNL had higher (or worse) levels of patient-reported symptom burden in relation to swallowing and eating solid foods. Like the PAS, FOIS and MASA-C scores, the aryepiglottic folds, pyriform sinus and cricopharyngeal prominence were again amongst some of the strongest predictors for symptom burden with solid foods.

## Discussion

This study describes the trajectory of external and internal HNL and its association with dysphagia in a homogeneous cohort of HNC patients treated with CRT. The results indicate that external and internal HNL are both prevalent following CRT and that two separate trajectory patterns exist in the 12&nbsp;months post-treatment. External HNL was most prevalent at 3 months, began to improve at 6 months and had largely resolved by 12&nbsp;months. In contrast, internal HNL persisted throughout the whole 12&nbsp;months and whilst there was some reduction in its severity and diffuseness, it never fully resolved during the study period. It can therefore be expected that patients with HNC who are 12&nbsp;months post-CRT may present with internal HNL in the absence of external HNL. Patients who also have a higher severity of external and/or internal HNL during the first 12&nbsp;months post-treatment, and those with more diffuse internal HNL may also experience more severe dysphagia.

The current study is consistent with previous research that has demonstrated the high prevalence of external and internal HNL in HNC patients post-treatment [6, 9, 10, 12, 19]. However, prior studies have examined both surgical and non-surgical treatment modalities which contrasts with the current study’s homogeneous cohort. However, the use of a homogeneous cohort comprising only of HNC patients treated with CRT can also be seen as a strength, in that it has provided evidence that these non-surgical patients are just as likely to develop external and internal HNL in the first 12 months post-treatment as their surgical counterparts. One difference though is that the peak severity of HNL in the current study occurred around 3 months, whereas it occurred around 9 months in Ridner’s et al. [6] longitudinal study. It is unknown whether this difference can be attributed their heterogeneous population, but it is a disparity that warrants further investigation.

The current study has also shown that whilst the presence of internal HNL remains constant across the 12 months following CRT, its presentation constantly changes. HNL that occurred at five of the 13 internal sites, including the aryepiglottic folds, valleculae and pyriform sinus, consistently improved from 3 to 12 months, whereas other internal sites, such as the epiglottis, showed early improvement between 3 and 6 months, and then plateaued from 6 to 12 months. In contrast, the arytenoids had limited improvement from 3 to 6 months and then significantly improved from 6 to 12 months. No other studies are currently available for comparison, but these results suggest that changes in HNL at individual internal sites are not uniform and that differences may exist in the lymphatic drainage of individual sites. Future studies that examine the trajectory of HNL at the individual internal sites, whilst also examining their lymphatic drainage patterns would be valuable.

It was also noted that whilst the general trend was for external and internal HNL to improve, some participants’ HNL worsened over the 12-month study period. It was noted that the most severe cases of external and internal HNL worsening occurred in participants with severe dysphagia who were either nil by mouth and tube dependent or restricted to a single food consistency. It is known that muscle contraction aides lymph transportation through the lymphatic vessels [34]; therefore, it may be postulated that the low frequency and force of swallows observed in those participants with severe dysphagia and particularly those who are tube dependent may further limit the effectiveness of the lymphatic system to drain stagnant lymph. This postulation warrants further longitudinal investigation in a larger sample size, but speech pathologists may need to consider the risk of none or limited oral intake for not only disuse atrophy and fibrosis, but also for worsening HNL.

Less than half of the participants in the current study who had external HNL at 3 months accessed treatment, despite being referred onto a free hospital service. These results suggest that some degree of spontaneous recovery has occurred as most participants’ external HNL resolved by 12&nbsp;months. However, significant improvements were not seen until after 6 months which may indicate that many participants lived with the burden of their external HNL for some time. Therefore, even though there may be some expectation of spontaneous recovery in HNC patients treated with CRT, timely access to external HNL treatment is still required.

The results of the current study also support the association between external and internal HNL and the presence of more severe dysphagia [7, 13, 16, 18, 19], and this association has now been demonstrated across acute, sub-acute and long-term periods post-HNC treatment. However, a limitation of the current evidence base is that none of the authors have described how external and internal HNL impact the physiological events that lead to penetration–aspiration risk and issues with bolus flow and clearance. It is logical to expect that patients with more severe and diffuse internal HNL would experience changes to their swallowing safety and efficiency [16], and the results of the current study would support this hypothesis. However, future studies that incorporate a more comprehensive assessment of swallowing function are required. In the meantime, speech pathologists and other oncologic clinicians need to be aware of the negative impact that external and internal HNL may have on swallowing function in HNC patients post-CRT. Laryngoscopy needs to be utilised to identify and measure internal HNL and monitor its progression and/or resolution over time.

The current study also raises questions about how internal and external HNL are measured. Patterson’s Radiotherapy Oedema Rating Scale does not utilise a composite score and this has led many authors [6, 9, 16, 18] to adopt the maximum severity score for internal HNL. However, this score may not reflect the change that occurs in internal HNL over time and may inflate its overall severity. For example, 64% of participants in this study were classified as having moderate or severe internal HNL at 12 months, but on average, only 2.2 of the 13 internal sites had moderate or severe HNL. The use of a sum severity score or the tallying of the number of internal sites affected, as undertaken in this study, may provide a more comprehensive overview of how internal HNL changes over time. The Patterson’s Radiotherapy Oedema Rating Scale was recently revised, now titled the Revised Patterson Oedema Scale [24], but there continues to be no guidance surrounding composite scoring.

The use of the MDACC Scale as an assessment for external HNL also has limitations. It is a clinical assessment only and hence lacks the sensitivity and psychometric testing of quantitative assessment tools, such as the ALOHA. The MDACC Scale was used as the primary assessment tool in the current study as at the time the study was designed, the ALOHA was not previously used in a diagnostic capacity and was only used to quantify the reduction of external HNL over time [20, 21]. However, normative TDC values for the head and neck region have recently been published in 2021 [22] and were incorporated in the analysis of the current study. The existence of normative reference data now improves the ALOHA’s capacity to be used as an isolated assessment tool.

## Limitations

It is acknowledged that the current study has several limitations. Firstly, a baseline or pre-treatment time point was sought, but participation was worse than expected and there was insufficient data to include in this study. This has been attributed to the high levels of anxiety and distress surrounding diagnosis and treatment. Similarly, participation in the 3-month time point was also low, with participants often declining this study appointment due to the recency of treatment and some anxiety surrounding their post-treatment PET-CT scan results. These factors, along with general attrition, may affect this study’s generalisability.

Secondly, the reliability of the current studies internal HNL ratings using Patterson’s Radiotherapy Oedema Rating Scale were also variable. The current study used an education package to support rating determinations which saw improved reliability compared to the authors’ previous work [10, 16], but further work in the area of clinician training is required. The reliability of the external HNL ratings was also not tested in this study.

Finally, there were several weaknesses in the FEES procedure, including no form of volumetric control with fluid trials, no food trials, no measure of pharyngeal residue and no description of physiological events. Other side effects that are known to influence swallowing and oral intake following HNC treatment, such as xerostomia, secretions and dysgeusia, were also not measured. The incorporation of these elements into future studies would be valuable to ensure that there is a more comprehensive assessment of swallowing function and diet status.

## Conclusion

External and internal HNL are both prevalent following CRT and two separate trajectory patterns exist in the 12&nbsp;months post-treatment. External HNL was most prevalent at 3 months, began to improve at 6 months and had largely resolved by 12&nbsp;months, whereas internal HNL persisted throughout the whole 12&nbsp;months, but with some changes to its severity and diffuseness. Patients who experienced higher severities of external and/or internal HNL and those who had more diffuse internal HNL also experienced more severe dysphagia. This study supports early screening, diagnosis and treatment of HNL post-treatment, but further consideration needs to be given to how internal HNL is treated and what role speech pathologists play in this management.

## References

[1] Sindhu SK, Bauman JE (2019). Current concepts in chemotherapy for head and neck cancer. Oral Maxillofac Surg Clin North Am.

[2] Pulte D, Brenner H (2010). Changes in survival in head and neck cancers in the late 20th and early 21st century: a period analysis. Oncologist.

[3] Barnhart MK (2018). Treatment toxicities and their impact on oral intake following non-surgical management for head and neck cancer: a 3-year longitudinal study. Support Care Cancer.

[4] Moroney LB (2018). Helical intensity-modulated radiotherapy with concurrent chemotherapy for oropharyngeal squamous cell carcinoma: a prospective investigation of acute swallowing and toxicity patterns. Head Neck.

[5] Miller MC, Shuman AG (2016). Survivorship in head and neck cancer: a primer. JAMA Otolaryngol Head Neck Surg.

[6] Ridner SH (2016). A prospective study of the lymphedema and fibrosis continuum in patients with head and neck cancer. Lymphat Res Biol.

[7] Murphy BA, Gilbert J, Ridner SH (2007). Systemic and global toxicities of head and neck treatment. Exp Rev Anticancer Therapy.

[8] Smith BG, Lewin JS (2010). Lymphedema management in head and neck cancer. Curr Opin Otolaryngol Head Neck Surg.

[9] Deng J (2012). Prevalence of secondary lymphedema in patients with head and neck cancer. J. Pain Symptom Manag..

[10] Jeans C (2020). Comparing the prevalence, location, and severity of head and neck lymphedema after postoperative radiotherapy for oral cavity cancers and definitive chemoradiotherapy for oropharyngeal, laryngeal, and hypopharyngeal cancers. Head Neck.

[11] Deng J (2012). Factors associated with external and internal lymphedema in patients with head-and-neck cancer. Int J Radiat Oncol Biol Phys.

[12] Tribius S (2020). Prognostic factors for lymphedema in patients with locally advanced head and neck cancer after combined radio(chemo)therapy- results of a longitudinal study. Oral Oncol.

[13] Deng J (2013). Impact of secondary lymphedema after head and neck cancer treatment on symptoms, functional status, and quality of life. Head Neck.

[14] Deng J (2012). Preliminary development of a lymphedema symptom assessment scale for patients with head and neck cancer. Support. Care Cancer.

[15] Nixon JL (2018). A mixed methods examination of distress and person-centred experience of head and neck lymphoedema. Oral Oncol.

[16] Jeans C (2021). Association between external and internal lymphedema and chronic dysphagia following head and neck cancer treatment. Head Neck.

[17] Jeans C (2019). Patient perceptions of living with head and neck lymphoedema and the impacts to swallowing, voice and speech function. Eur J Cancer Care (Engl).

[18] Jackson LK (2016). Internal lymphedema correlates with subjective and objective measures of dysphagia in head and neck cancer patients. J Palliat Med.

[19] Queija DDS (2020). Cervicofacial and pharyngolaryngeal lymphedema and deglutition after head and neck cancer treatment. Dysphagia.

[20] Nixon JL (2014). Pilot study of an assessment tool for measuring head and neck lymphoedema. Br J Community Nurs.

[21] Purcell A (2014). Measuring head and neck lymphedema: the "ALOHA" trial. Head Neck.

[22] Mayrovitz HN (2021). An approach toward assessing head-and-neck lymphedema using tissue dielectric constant ratios: method and normal reference values. Lymphat Res Biol.

[23] Patterson JM, Hildreth A, Wilson JA (2007). Measuring edema in irradiated head and neck cancer patients. Ann Otol Rhinol Laryngol.

[24] Starmer HM (2021). Development and reliability of the revised Patterson Edema scale. Clin Otolaryngol.

[25] Carnaby GD, Crary MA (2014). Development and validation of a cancer-specific swallowing assessment tool: MASA-C. Support Care Cancer.

[26] Crary MA, Mann GD, Groher ME (2005). Initial psychometric assessment of a functional oral intake scale for dysphagia in stroke patients. Arch Phys Med Rehabil.

[27] Rosenbek JC (1996). A penetration-aspiration scale. Dysphagia.

[28] Steele CM, Grace-Martin K (2017). Reflections on clinical and statistical use of the penetration-aspiration scale. Dysphagia.

[29] Cooperstein E (2012). Vanderbilt head and neck symptom survey version 2.0: report of the development and initial testing of a subscale for assessment of oral health. Head Neck.

[30] StataCorp (2015). Stata statistical software: release 15.

[31] Vanbelle S (2016). A new interpretation of the weighted kappa coefficients. Psychometrika.

[32] Landis JR, Koch GG (1977). The measurement of observer agreement for categorical data. Biometrics.

[33] Williams RL (2000). A note on robust variance estimation for cluster-correlated data. Biometrics.

[34] Reddy NP (1986). Lymph circulation: physiology, pharmacology, and biomechanics. Crit Rev Biomed Eng.

